# Identification of a chromatin regulator signature and potential candidate drugs for bladder cancer

**DOI:** 10.1186/s41065-021-00212-x

**Published:** 2022-02-07

**Authors:** Ke Zhu, Xiaoqiang Liu, Wen Deng, Gongxian Wang, Bin Fu

**Affiliations:** 1 Department of Urology, The First Affiliated Hospital of Nanchang University, 17 Yongwaizheng Street, Nanchang, Jiangxi 330006 P.R. China; 2 Jiangxi Institute of Urology, Nanchang, Jiangxi 330006 PR China

**Keywords:** Chromatin regulators, Bladder cancer, TCGA, Prognosis

## Abstract

**Background:**

Bladder cancer (BLCA) is a malignant tumor with a dismay outcome. Increasing evidence has confirmed that chromatin regulators (CRs) are involved in cancer progression. Therefore, we aimed to explore the function and prognostic value of CRs in BLCA patients.

**Methods:**

Chromatin regulators (CRs) were acquired from the previous top research. The mRNA expression and clinical information were downloaded from TCGA and GEO datasets. Cox regression analysis and least absolute shrinkage and selection operator (LASSO) regression analysis were performed to select the prognostic gene and construct the risk model for predicting outcome in BLCA. The Kaplan-Meier analysis was used to assess the prognosis between high- and low-risk groups. We also investigated the drug sensitivity difference between high- and low-risk groups. CMAP dataset was performed to screen the small molecule drugs for treatment.

**Results:**

We successfully constructed and validated an 11 CRs-based model for predicting the prognosis of patients with BLCA. Moreover, we also found 11 CRs-based model was an independent prognostic factor. Functional analysis suggested that CRs were mainly enriched in cancer-related signaling pathways. The CR-based model was also correlated with immune cells infiltration and immune checkpoint. Patients in the high-risk group were more sensitive to several drugs, such as mitomycin C, gemcitabine, cisplatin. Eight small molecule drugs could be beneficial to treatment for BLCA patients.

**Conclusion:**

In conclusion, our study provided novel insights into the function of CRs in BLCA. We identified a reliable prognostic biomarker for the survival of patients with BLCA.

**Supplementary Information:**

The online version contains supplementary material available at 10.1186/s41065-021-00212-x.

## Introduction

Bladder cancer (BLCA), as a worldwide health issue, is one of the malignancies with significant sex discriminations that incidence and mortality are approximately 4 times higher among men than in women [1]. Despite encouraging achievements being accomplished in diagnosis and treatment, including liquid biopsy, targeted therapy, and immunotherapy, the overall survival of BLCA patients remains to be unsatisfactory [2–9]. Accumulating evidence has confirmed that multigene signature could offer risk stratification and prognostic prediction in cancer [10–14]. Therefore, this research aims to build a chromatin regulator signature to forecast the overall survival of patients with BLCA.

Epigenetic alterations, which were considered as one of the most significant hallmarks of tumors, were actuated by chromatin regulators (CRs). CRs were indispensable regulatory elements of epigenetics [15]. CRs were mainly classified into three categories based on roles in epigenetics: DNA methylators, histone modifiers, and chromatin remodelers [16]. But these three categories were closely associated with each other when involved in biological processes. Further studies have revealed that the aberrant expressions of CRs were implicated in multiple biological processes including inflammation [17], apoptosis [18], autophagy [19], and proliferation [20], which indicated that CRs deregulation could result in the development of many diseases including cancer. CRs have been proved to be abnormally expressed and associated with outcomes across cancer types. HMGA1, belonging to chromatin remodeler, was obviously elevated in BLCA, and HMGA1 deletion attenuated the proliferation, invasion, and activated the autophagy by regulating miR-221/TP53INP1/p-ERK1/2 pathway [21]. CBX7, a member of chromobox family with epigenetic regulation, has been found to be downregulated in BLCA, especially in patients with high grade, advanced T and N stage [22]. Furthermore, the overexpression of CBX7 was correlated with longer survival. CBX7 overexpression inhibited the malignant progression of BLCA cells via transcriptional regulating AKR1B10/ERK signaling. WDR5, a crucial member of the MLL/SET1 complexes with methyltransferase activity, has been reported to be markedly upregulated in BLCA, and the overexpression of WDR5 was associated with advanced tumor stage and shorter survival [23]. Further *in vitro and in vivo* experiments suggested that WDR5 promoted tumor growth and enhanced the chemoresistance of tumor cells to cisplatin. ZNF671, as a DNA methylator, inhibited cell growth and invasion in BLCA [24]. EZH2, a histone lysine methyltransferase, was implicated in diverse malignant phenotypes such as apoptosis and metastasis in multiple types of cancers [25–27].

A better understanding of CRs is crucial for BLCA progression and therapy, as well as paving the way for further research. However, few pieces of researches have been systematically explored the relationship between CRs and BLCA. In this study, we focused on investigating the expression profiles and prognostic values of CRs in BLCA through bioinformatic analysis. We successfully constructed and proved a prognostic signature based on 11 CRs, which could effectively predict the outcome of BLCA patients. Furthermore, we found the relationship between the prognostic signature and the immune microenvironment in BLCA, providing a theoretical basis for the immune checkpoint therapy strategies. In addition, we also found 8 small molecule drugs that might be beneficial for treatments of BLCA patients.

## Materials and Methods

### Data collection and identification of differentially expressed CRs

The mRNA expression and relevant clinical information of 19 normal bladder tissues and 414 bladder cancer tissues were obtained from the public database (the Cancer Genome Atlas, TCGA, https://portal.gdc.cancer.gov). A total of 870 Chromatin regulators (CRs) were retrieved from previous topic research [15]. We also downloaded GSE13507 [28] consisting of 165 bladder cancer tissues from a public database (Gene Expression Omnibus, GEO, https://www.ncbi.nlm.nih.gov/geo/) for the validation. These mRNA expression profiles were normalized through the corresponding R package. According to the criteria of |logFC| >1 and false discovery rate (*FDR)* < 0.05, differentially expressed CRs were identified by limma package based on R software.

### Functional enrichment analyses and protein-protein interaction (PPI)

Gene Ontology (GO) analysis, including molecular function (MF), biologic process (BP), and cellular components (CC), was performed through the ClusterProfiler package. The Kyoto Encyclopedia of Genes and Genomes (KEGG) pathway analysis was conducted with the same method. FDR and *p* < 0.05 were regarded as significantly enriched. We submitted the differently expressed CRs to the STRING database (http://www.string-db.org/) to obtain protein-protein interaction information. Cytoscape software was used to construct and visualize the PPI network. We implemented the MCODE plug-in to select the most significant module of the PPI network by MCODE scores > 10.

### Screening for potential small molecule drugs

We uploaded differently expressed CRs into the Connectivity MAP database (CMAP, https://portals.broadinstitute.org/cmap/) to screen potential small molecule drugs related with CRs for treatments of BLCA patients. Scores were set from −1 to 1 to assess the degree of closeness in the compound-related with uploaded genes. Drugs with negative scores could exert the anti-cancer function. And the set threshold was *p* < 0.01, n ≥ 3, percent non-null =100 and enrichment < -0.8.

### Construction and validation of a prognostic model based on CRs

We performed univariate Cox regression analysis to further screen the prognostic value of CRs. Then lasso-penalized Cox regression analysis were utilized to construct the prognostic risk model through glmnet R package. Risk scores were calculated by the following tool:

$$\mathrm{Risk}\ \mathrm{score}=\left(\mathrm{Coef}1\ast \mathrm{expression}\ \mathrm{mRNA}1\right)+\left(\mathrm{Coef}2\ast \mathrm{expression}\ \mathrm{mRNA}2\right)+\left(\mathrm{Coef}\ \mathrm{n}\ast \mathrm{expression}\ \mathrm{mRNA}\ \mathrm{n}\right)$$

where Coef is lasso Cox regression model coefficient of the corresponding mRNA. We divided BLCA patients into high-risk groups and low-risk groups in accordance with the median risk score. Survival analysis was performed by using Kaplan-Meier curve to evaluate the prognosis in two groups. Time-related ROC analysis was applied to assess the prognostic ability of the risk model via survivalROC package. A GEO dataset was considered as the validated set for further verification of the prognostic performance of this model.

### Gene Set Enrichment Analysis (GSEA)

To investigate the underlying molecular mechanisms among low- and high-risk groups, Gene Set Enrichment Analysis (GSEA) analysis was conducted. *P* value < 0.05 and FDR < 25% were considered statistically significant.

### Nomogram establishment based on risk score and clinical variables

We researched the relationship between CR-based signature and Clinical variables. In addition, combined with other clinical variables, we performed univariate and multivariate Cox regression analyses for exploring whether risk scores had an independent prognostic value in BLAC patients. Clinical variables and the CR-based signature risk score were applied to establish a nomogram associated with outcome for evaluating the probability of 3-, and 5-year OS for BLCA patients. The concordance index (C-index) and calibration curve were performed to assess the predictive utility of the nomogram.

### Immune cell infiltration analysis

Mounting research confirmed that tumor cells immune infiltration was involved in

cancer progression and correlated with prognosis. Therefore, we evaluated the infiltration level of immune cells between high-risk groups and low-risk groups based on B cell-specific lncRNA signature by using CIBERSORT, CIBERSORT-ABS, QUANTISEQ, MCP-counter, XCELL, TIMER, and EPIC algorithms. In order to predict the effect of immune checkpoint blockade therapy, we also explored the expression of several immune checkpoints such as PDCD1, HAVCR2 (Tim-3), PD-L1, LAG3, TIGIT, and CTLA4. In addition, the TIMER database (https://cistrome.shinyapps.io/timer/) was used to identify the relationship between immune cells and 11 CRs and improve our understanding of the role of CRs in BLCA.

### Drug sensitivity analysis

To explore the sensitivity difference of drugs between two groups, we used the Genomics of Drug Sensitivity in Cancer (GDSC, http://www.cancerrxgene.org/) database to analyze the half-maximal inhibitory concentration (IC50) of drugs for predicting the drug sensitivity by using the package (pRRophetic). *P* values < 0.05 were considered statistically significant.

### Statistics analysis

All statistical analyses were conducted by R software (version 4.0.5). The Wilcoxon test was used to compare the differences among the two groups. *P*-value < 0.05 was considered statistically significant.

## Results

### Establishment and validation of CR-based signature

**Figure 1:**
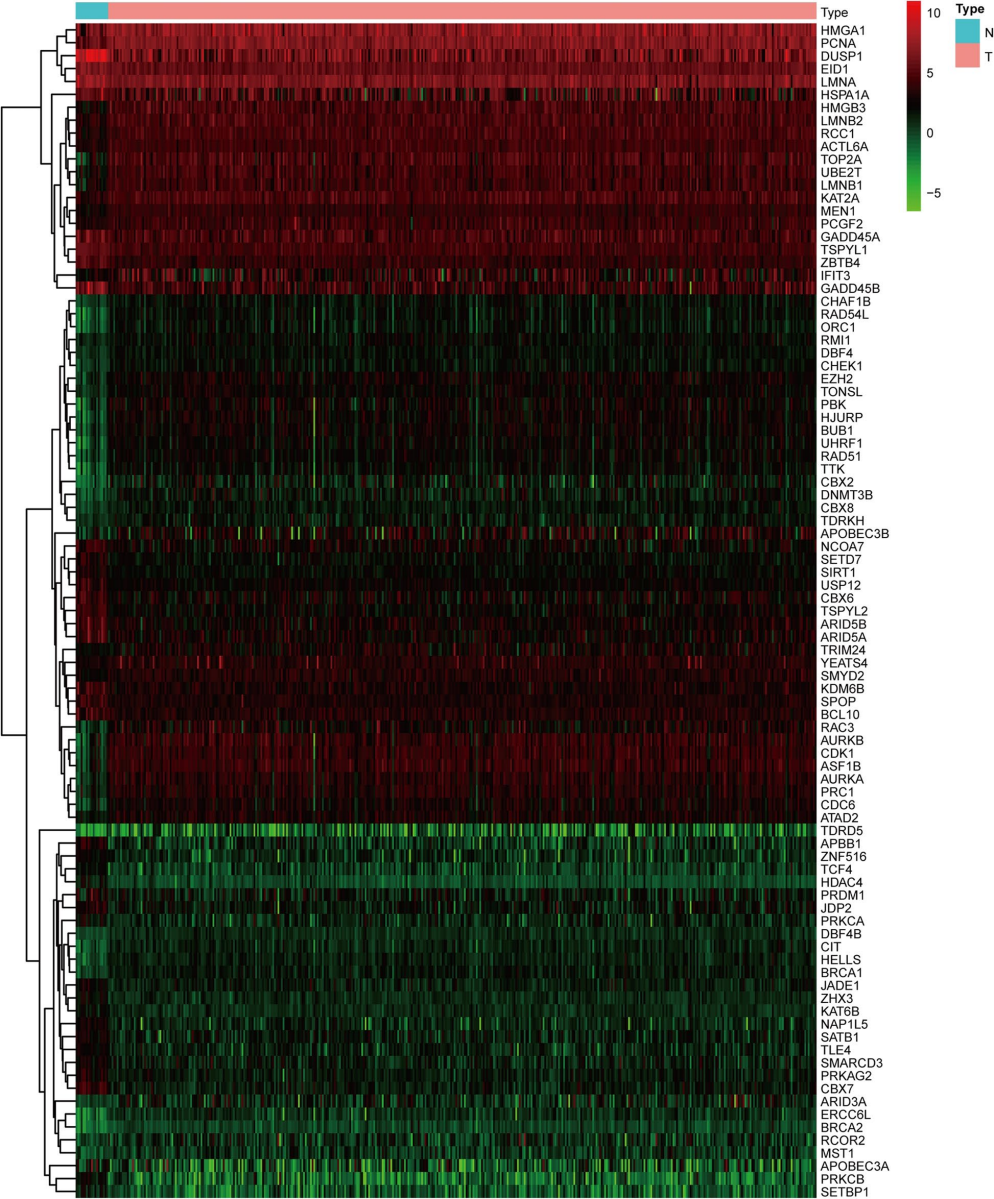
Heatmap showed differentially expressed CRs

**Figure 2:**
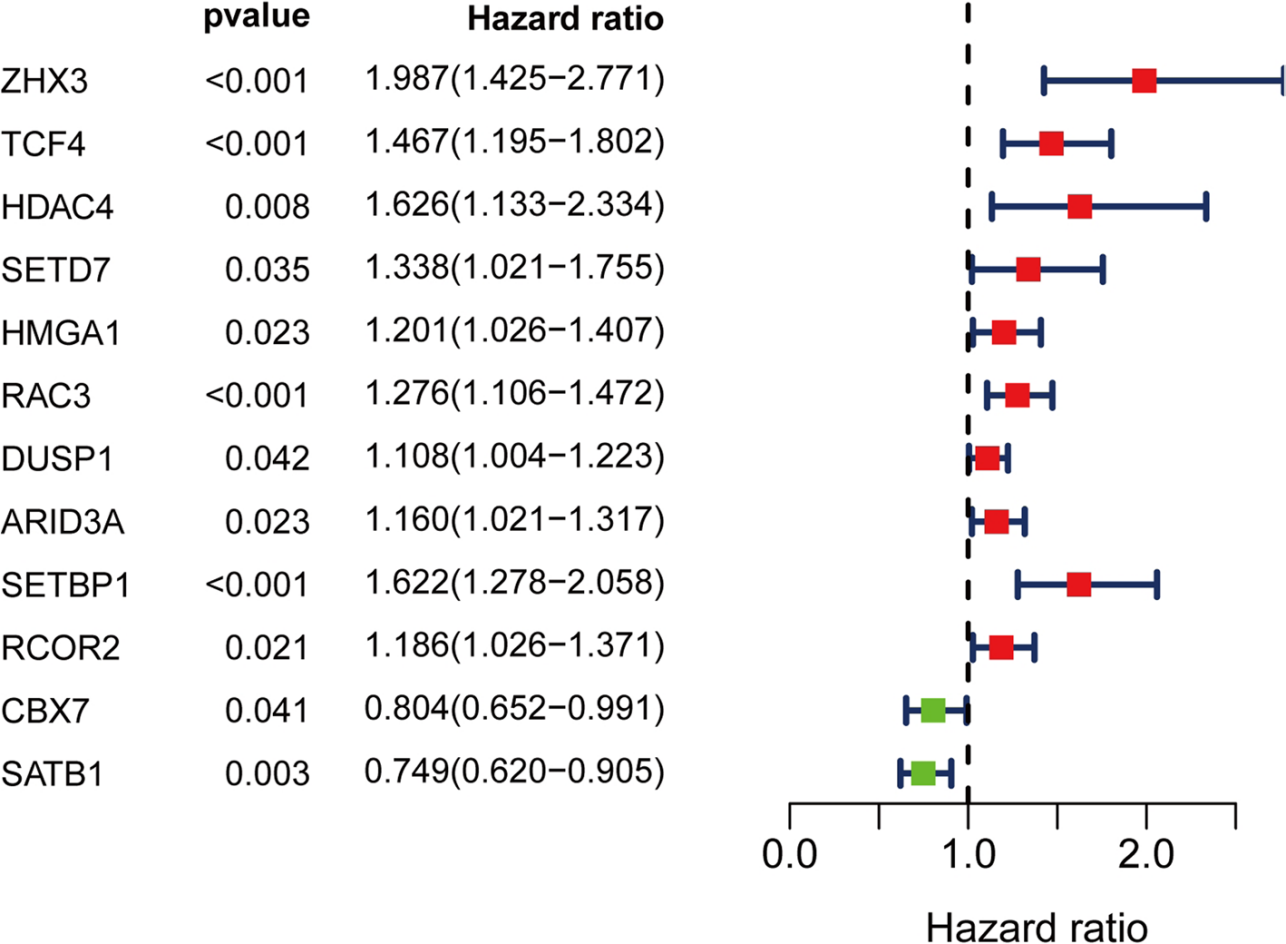
Identification of prognostic CRs by univariate Cox regression analysis

**Figure 3:**
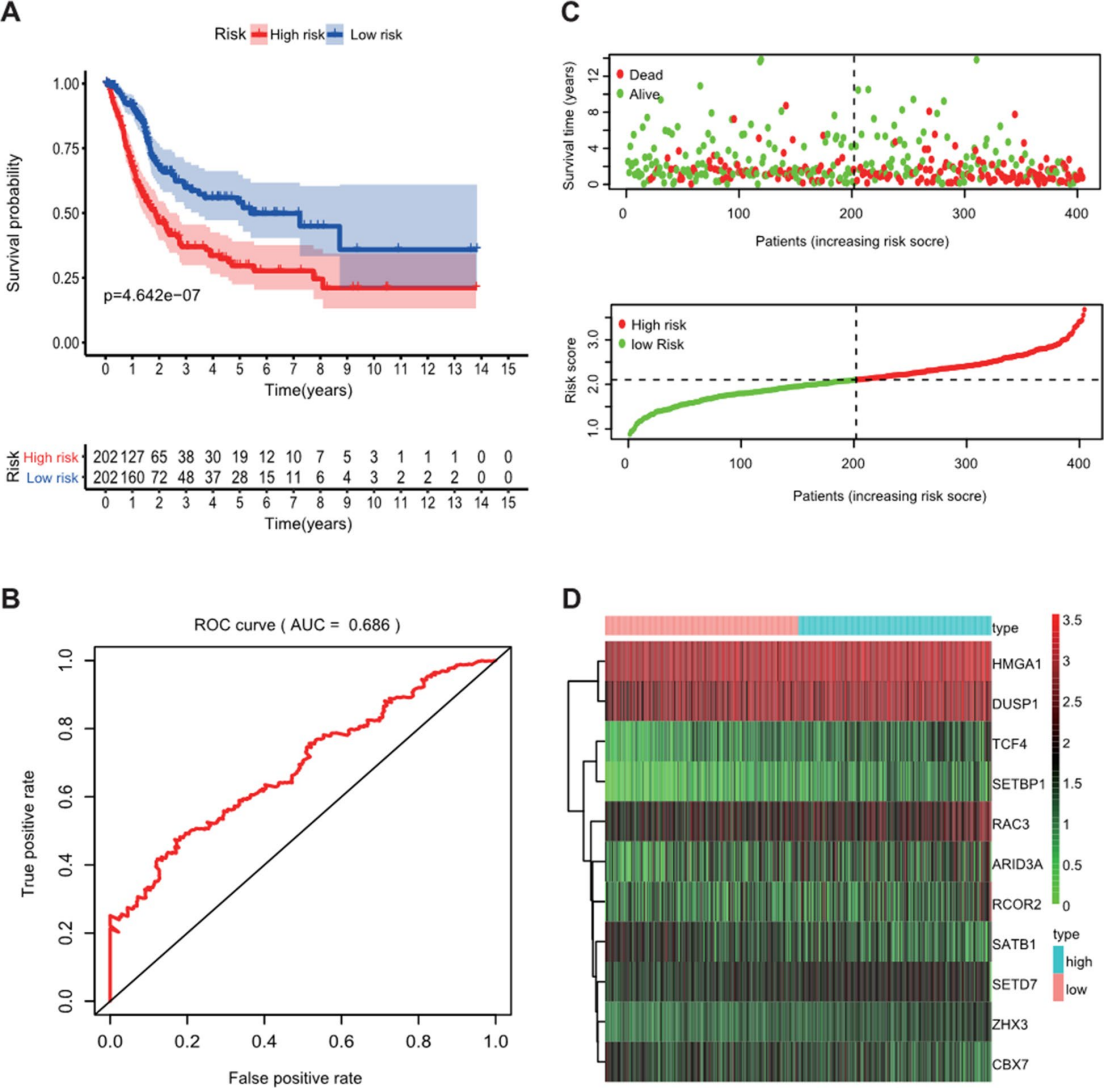
Construction of the prognostic CR-based signature in TCGA set. **A**. Kaplan-Meier survival analysis of BLCA patients between high-risk groups and low-risk groups in TCGA set; **B**. Time-independent receiver operating characteristic (ROC) analysis of risk scores predicting the overall survival in TCGA set; **C.** Distribution of survival status based on the median risk score in TCGA set; **D**. Heatmap showed the differences of 11 chromatin regulators between high and low-risk patients in TCGA set

**Figure 4:**
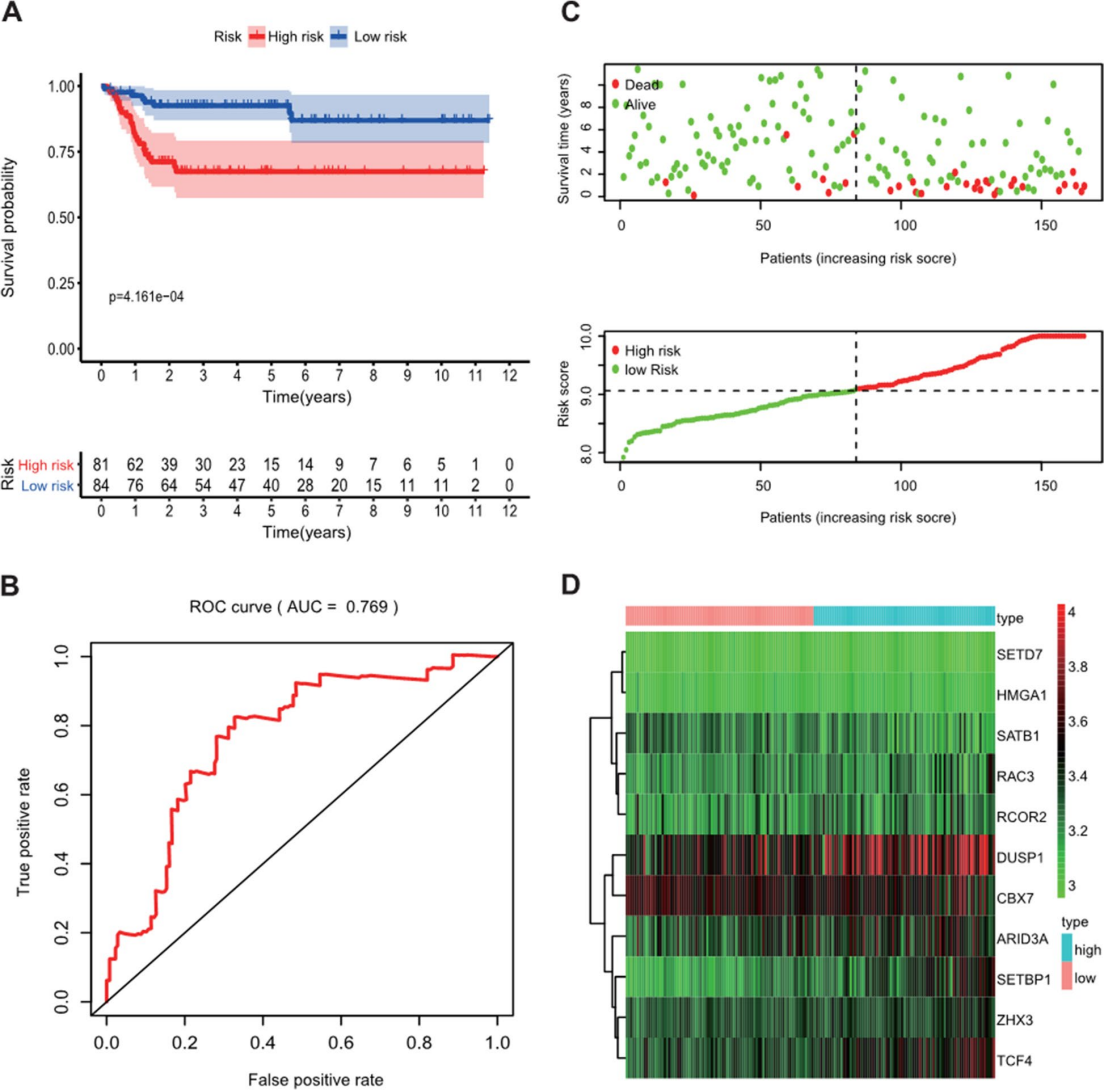
Validation of the prognostic CR-based signature in the GSE13507 set. **A**. Kaplan-Meier survival analysis of BLCA patients between high-risk groups and low-risk groups in GSE13507 set; **B**. Time-independent receiver operating characteristic (ROC) analysis of risk scores predicting the overall survival in GSE13507 set; **C**. Distribution of survival status based on the median risk score in GSE13507 set; **D**. Heatmap showed the differences of 11 chromatin regulators between high and low-risk patients in GSE13507 set

Compared with the normal bladder tissues, a total of 91 CRs, including 38 down-regulated CRs and 53 up-regulated CRs, were identified as differently expressed CRs in the TCGA-BLCA dataset (Figure 1). Based on these deregulated CR, we used univariate Cox regression analysis to explore the prognostic value of CR. The result showed that only 12 of them had prognostic merit (Figure 2). Then, LASSO Cox regression analysis was used to construct a signature for prognostic prediction capacity of BLCA patients. A risk model was successfully constructed with 11 genes (ZHX3, TCF4, SETD7, HMGA1, ARID3A, RAC3, DUSP1, SETBP1, CBX7, RCOR2, and SATB1). The risk score was calculated by relevant coefficients of 11 CRs as following formula (Table 1): risk score= (0.3622 × ZHX3 expression) +(0.1749 × TCF4 expression) + (0.1362 × SETD7 expression) + (0.0711 × HMGA1 expression) + (0.1523 × RAC3 expression) + (0.0217 × DUSP1 expression) + (0.0721 × ARID3A expression) + (0.2859 × SETBP1 expression) + (0.0979 × RCOR2 expression) + (-0.1999 × CBX7 expression) + (-0.881 × SATB1 expression). BLCA patients were classified into two groups (high-risk groups and low-risk groups) in line with the median risk score. The deaths of the high-risk group were significantly more than those of the low-risk group (*p* < 0.001), which suggested that risk score was negatively correlated with prognosis (Figure 3A, and C). The time-dependent ROC analysis showed that the prognostic accuracy of the CR-based signature in the TCGA dataset was 0.686 at 5-year (Figure 3B). We also validated the prognostic value of the CR-based signature in the GEO dataset in the same method. The result was consistent with the TCGA dataset (Figure 4A, and C). The time-dependent ROC curve showed the AUCs was 0.769 at 5-years (Figure 4B).

**Table 1:** Gene list and coefficient

Gene symbol	coefficient
ZHX3	0.3622
TCF4	0.1749
SETD7	0.1362
HMGA1	0.0711
RAC3	0.1523
DUSP1	0.0217
SETBP1	0.2859
RCOR2	0.0979
SATB1	-0.0881
ARID3A	0.0721
CBX7	-0.1999

### The CR-based signature was an independent indicator of BLCA prognosis

**Figure 5:**
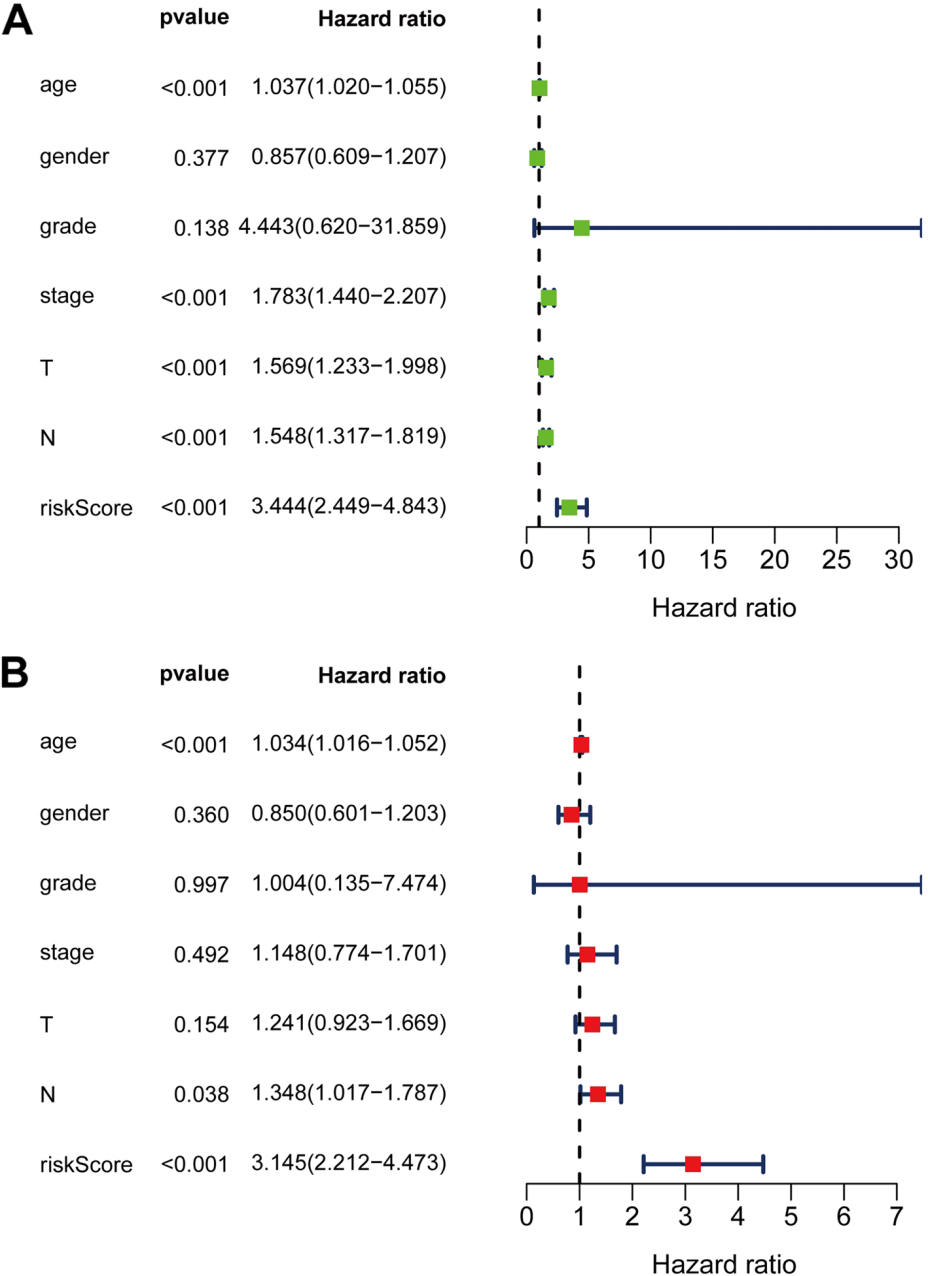
The signature was an independent prognostic factor for BLCA in the TCGA set. (**A**) The correlations between the risk score for OS and clinicopathological factors by univariate Cox regression analysis; (**B**) The correlations between the risk score for OS and clinicopathological factors by multivariate Cox regression analysis

We executed univariable and multivariable Cox analyses to testify whether this signature could be an independent prognostic indicator. Univariate analysis showed that risk score, TNM stage, N stage, T stage, and age were significantly relevant to the survival of BLCA patients (*p* < 0.001) (Figure 5A). Multivariate analysis indicated that the risk score, N stage, and age were still remarkably related to prognosis (*p* < 0.05) (Figure 5B). These results demonstrated that CR-based signature was an independent prognostic indicator for BLCA patients.

### Association between the signature and clinical characteristics

**Figure 6:**
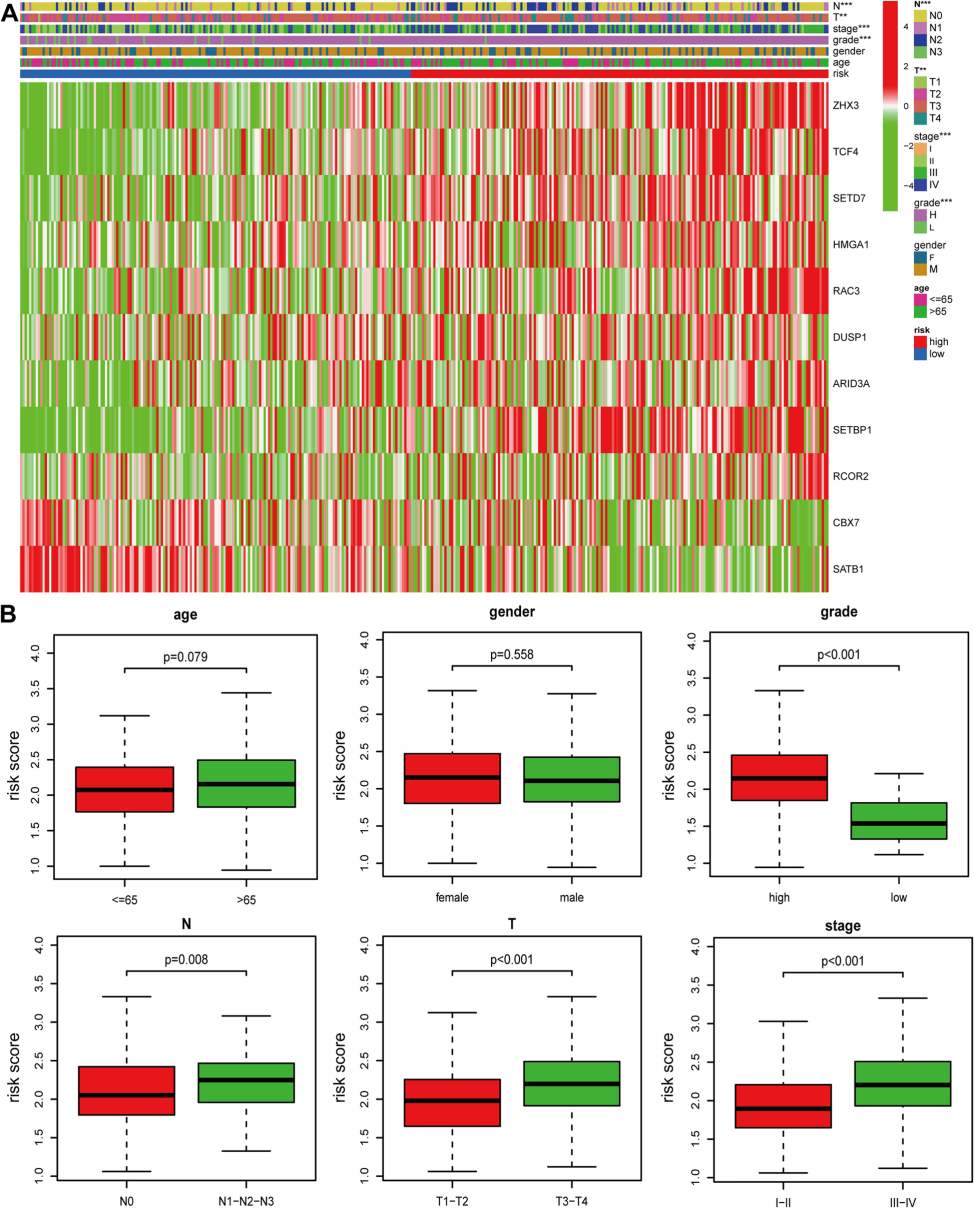
Correlation between signature and clinical characteristics

**Figure 7:**
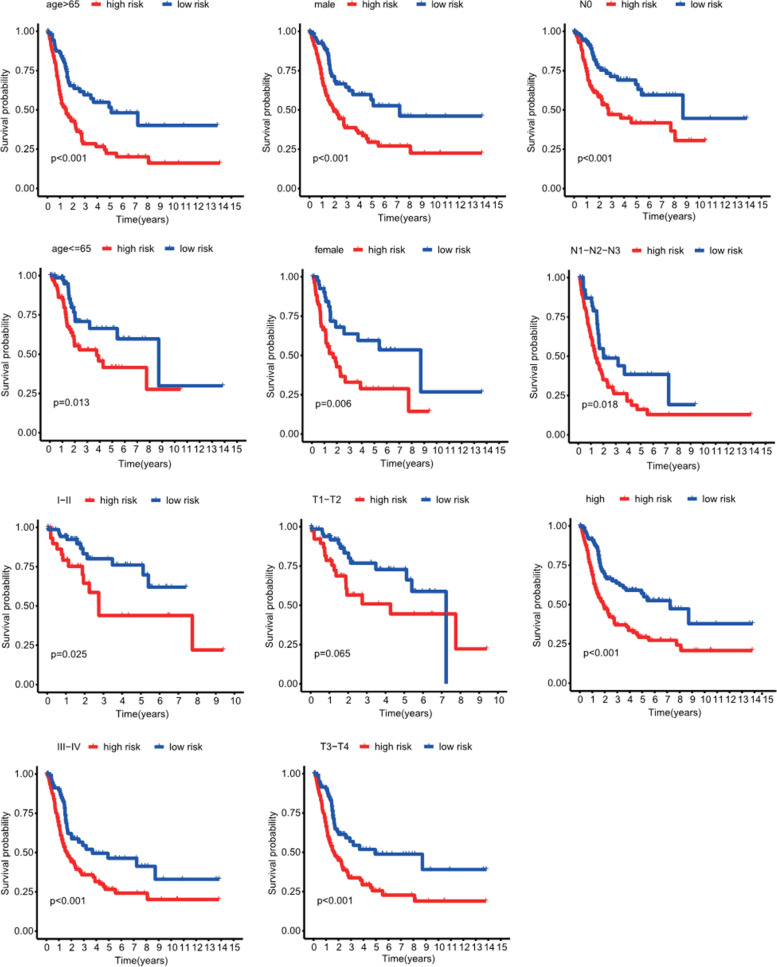
Kaplan-Meier curves of OS differences stratified by gender, age, grade, N stage, T stage, or TNM stage between the high-risk groups and low-risk groups

Chi-square test was used to explore whether the prognostic signature participated in the development and progression of BLCA. The result (Figure 6A-B) showed that there were significant differences between high- and low-risk groups in tumor grade (*p* < 0.001), TNM stage (*p* < 0.001), N stage (*p* < 0.01), and T stage (*p <* 0.001) but no significant differences in age and gender (*p >* 0.05). Moreover, stratification analysis was further conducted to investigated the prognostic significance of the signature in subgroups. Our research suggested that CR-based signature showed excellent performance in predicting outcome in age > 65 (*p* < 0.001), age <=65 (*p* = 0.013), female (*p* < 0.01), male (*p* < 0.001), Stage I-II (*p* = 0.025), Stage III-IV (*p* < 0.001), Grade high (*p* < 0.001), T3-T4 stage (*p* < 0.001), N0 (*p* < 0.001) and N1-2-3 (*p* = 0.018). While CR-based signature showed poor performance in predicting outcome in T1-T2 stage and Grade low (*p* > 0.05) (Figure 7).

### Construction of a Nomogram

**Figure 8:**
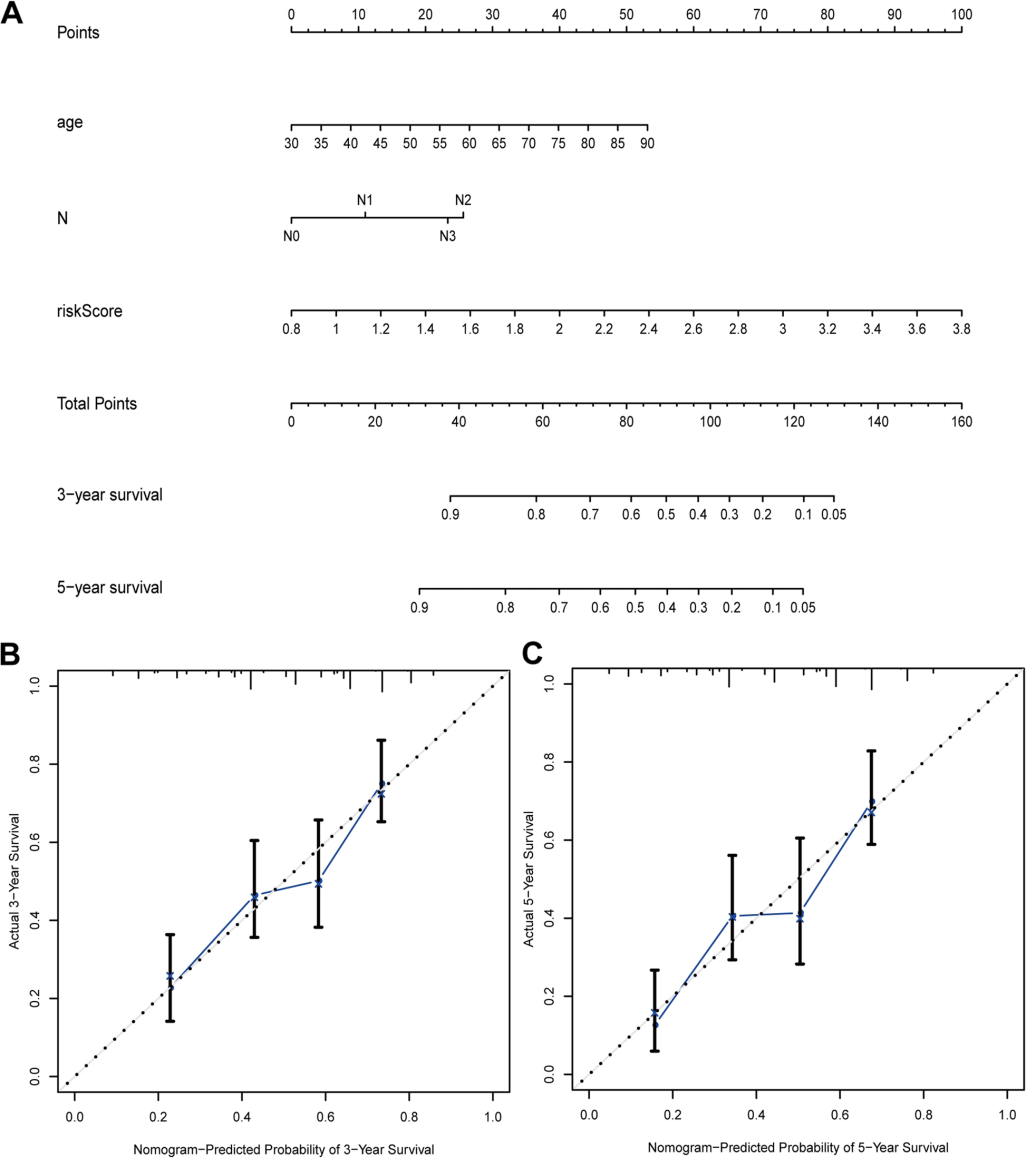
Construction of a nomogram. (**A**) nomogram for predicting 3- or 5-year OS; (**B**) The calibration plots for predicting 3-year OS; (**C**) The calibration plots for predicting 5-year OS

The nomogram incorporated various prognostic indicators to assess the survival probability of an individual graphically. To further forecast the survival of BLCA patients, we structured a nomogram comprised of N and risk score as well as age. Nomography predicted the 3-, 5-year survival rate of patients with BLCA (Figure 8A). The calibration curve indicated that the practical survival of the patient was in line with the predicted value (Figure 8B-C). The C index of the nomogram was 0.691, which confirmed the favorable prediction ability of the nomogram.

### Functional enrichment analyses and Protein-Protein interaction (PPI)

**Figure 9:**
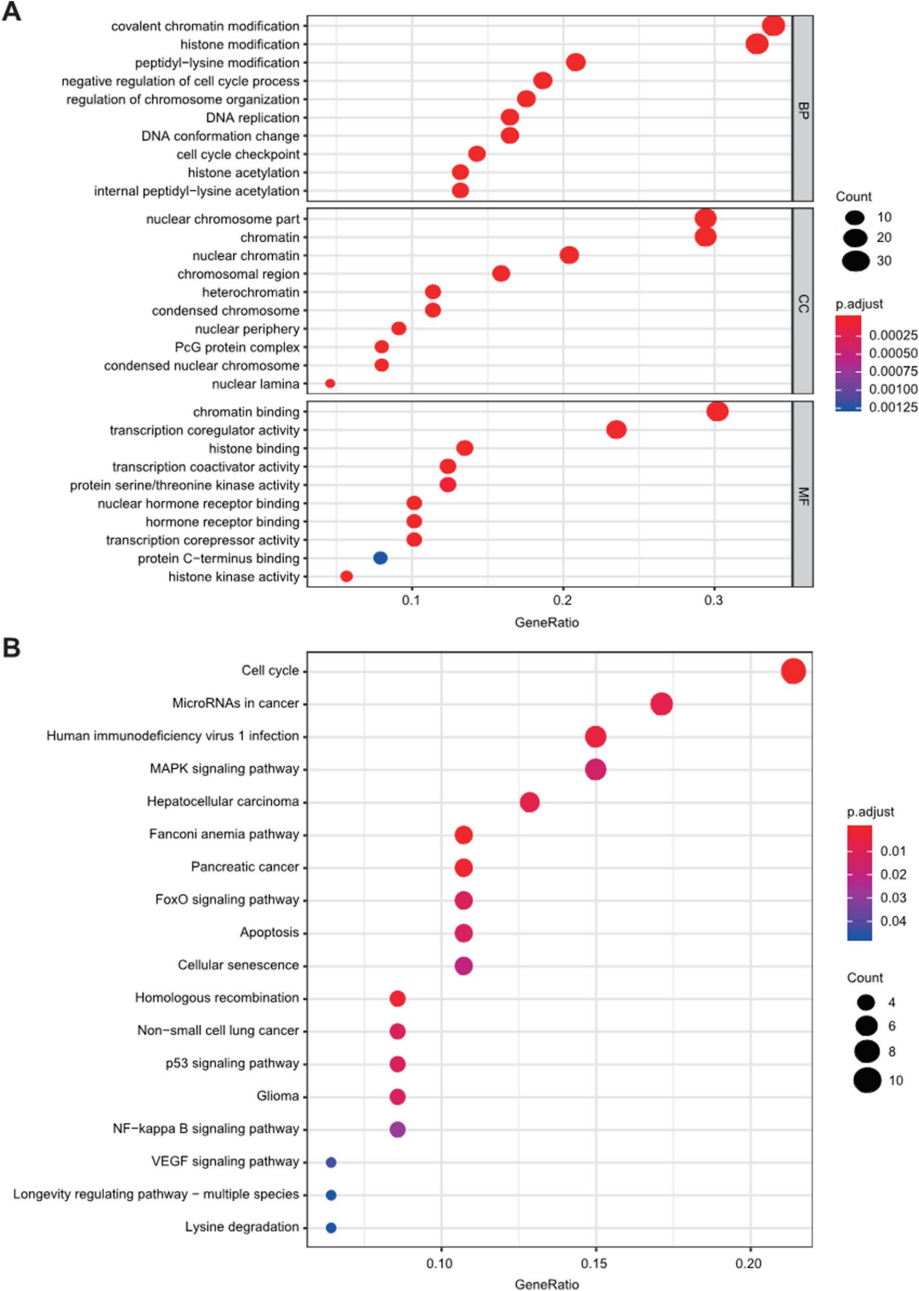
Enrichment analyses of differentially expressed CRs. (**A**) GO analysis; (**B**) KEGG analysis

**Figure 10:**
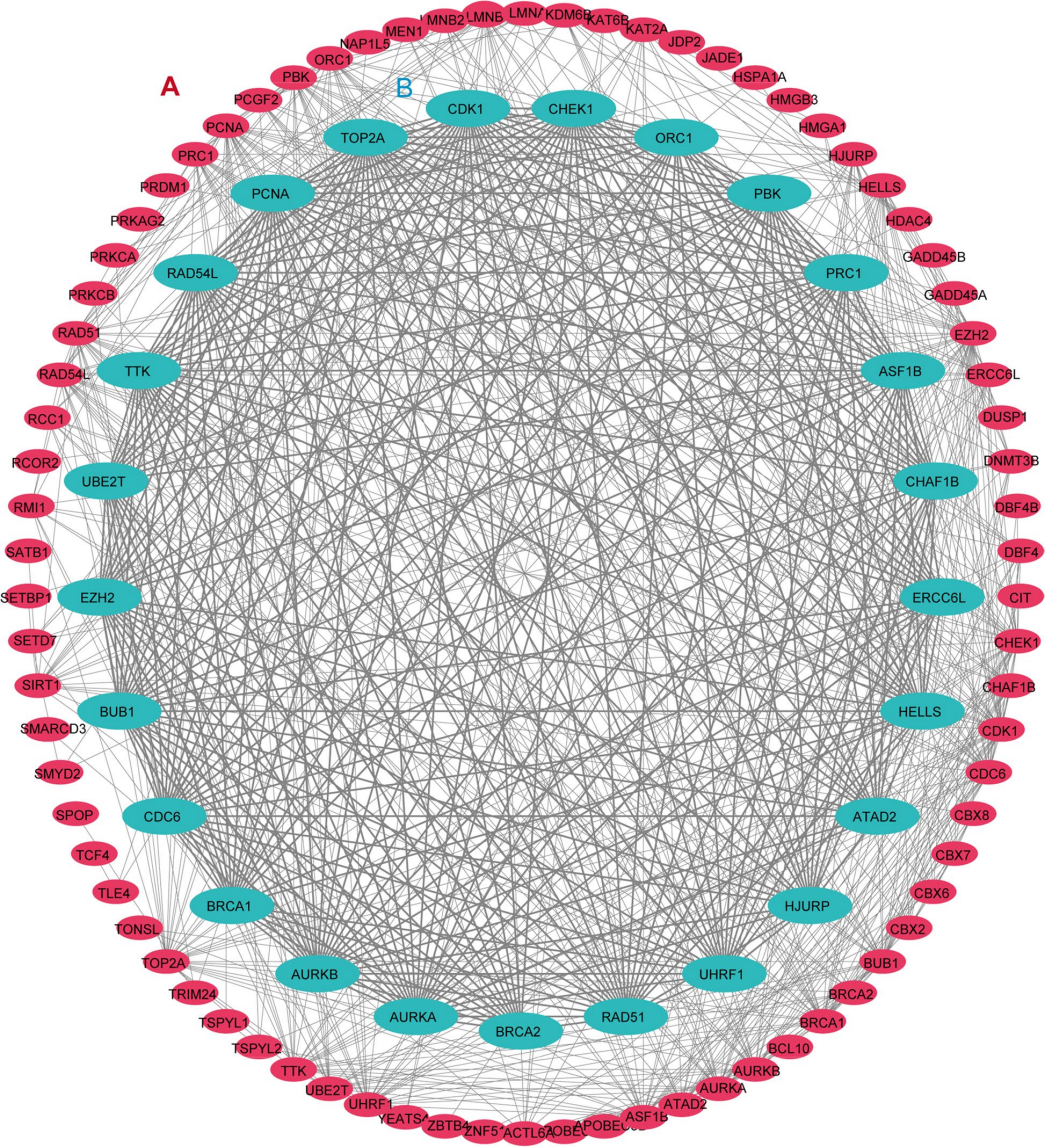
Protein-protein interaction network of differentially expressed CRs

GO and KEGG analyses were carried out to explore the potential function of differently expressed CRs. The results of biological process analyses showed that 91 CRs were remarkablly involved in covalent chromatin modification, histone modification, DNA replication, and peptidyl lysine modification. Cellular component analysis suggested that nuclear chromosome part, chromatin, heterochromatin, chromosomal region, and PcG protein complex were mainly enriched. Molecular function analysis indicated that 91 CRs were majorly located in chromatin binding, transcription coregulator activity, histone binding, and nuclear hormone receptor binding (Figure 9A). In the KEGG pathways, the results indicated that these genes were mainly involved in the cell cycle, microRNAs in cancer, apoptosis, p53 signaling pathway, MAPK signaling pathway, and FoxO signaling pathway (Figure 9B). STRING database showed that the PPI network of the differentially expressed CRs comprised 82 nodes and 479 edges (Figure 10A). The most meaningful module consisted of 25 CRs, including 25 nodes and 266 edges (Figure 10B).

### GSEA

**Figure 11:**
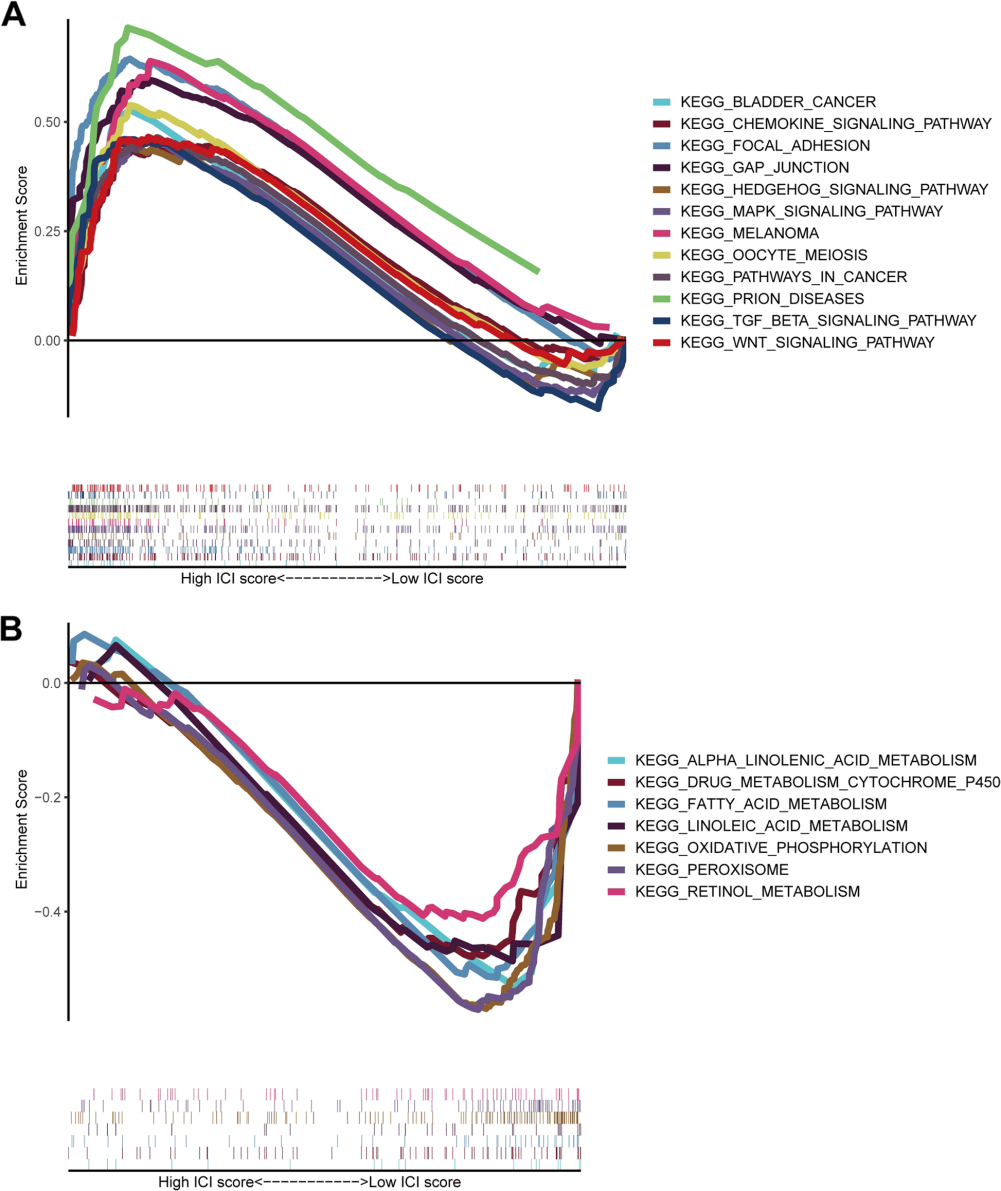
GSEA analysis

To further elucidate the molecular mechanisms of CR-based signature, GSEA analysis was performed. The results of GSEA analysis showed that bladder cancer, pathways in cancer, focal adhesion, GAP Junction, oocyte meiosis, chemokine signaling pathway, melanoma, WNT signaling pathway, prion disease, TGF-β signaling pathway, Hedgehog signaling pathway, and MAPK signaling pathway were mainly enriched in the high-risk group, while alpha-linolenic acid metabolism, drug metabolism cytochrome P450, oxidative phosphorylation, fatty acid metabolism, peroxisome, and retinol metabolism were mainly enriched in low-risk group (Figure 11).

### Immune infiltration level analysis of the CR-based signature

**Figure 12:**
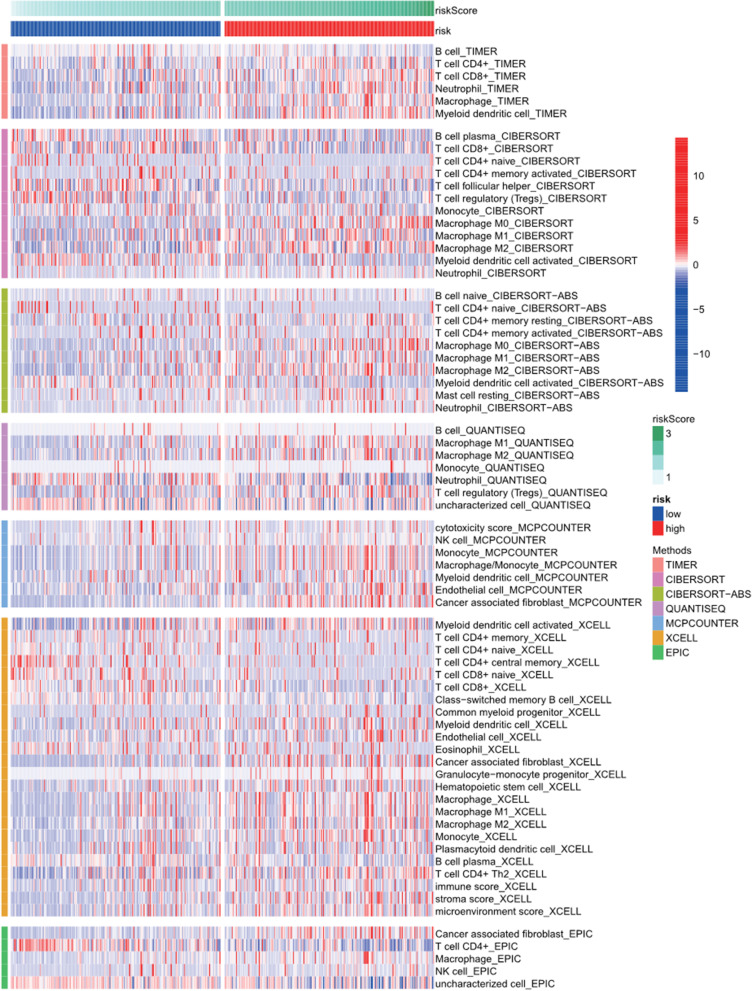
Immune cells infiltration between high-risk groups and low-risk groups

**Figure 13:**
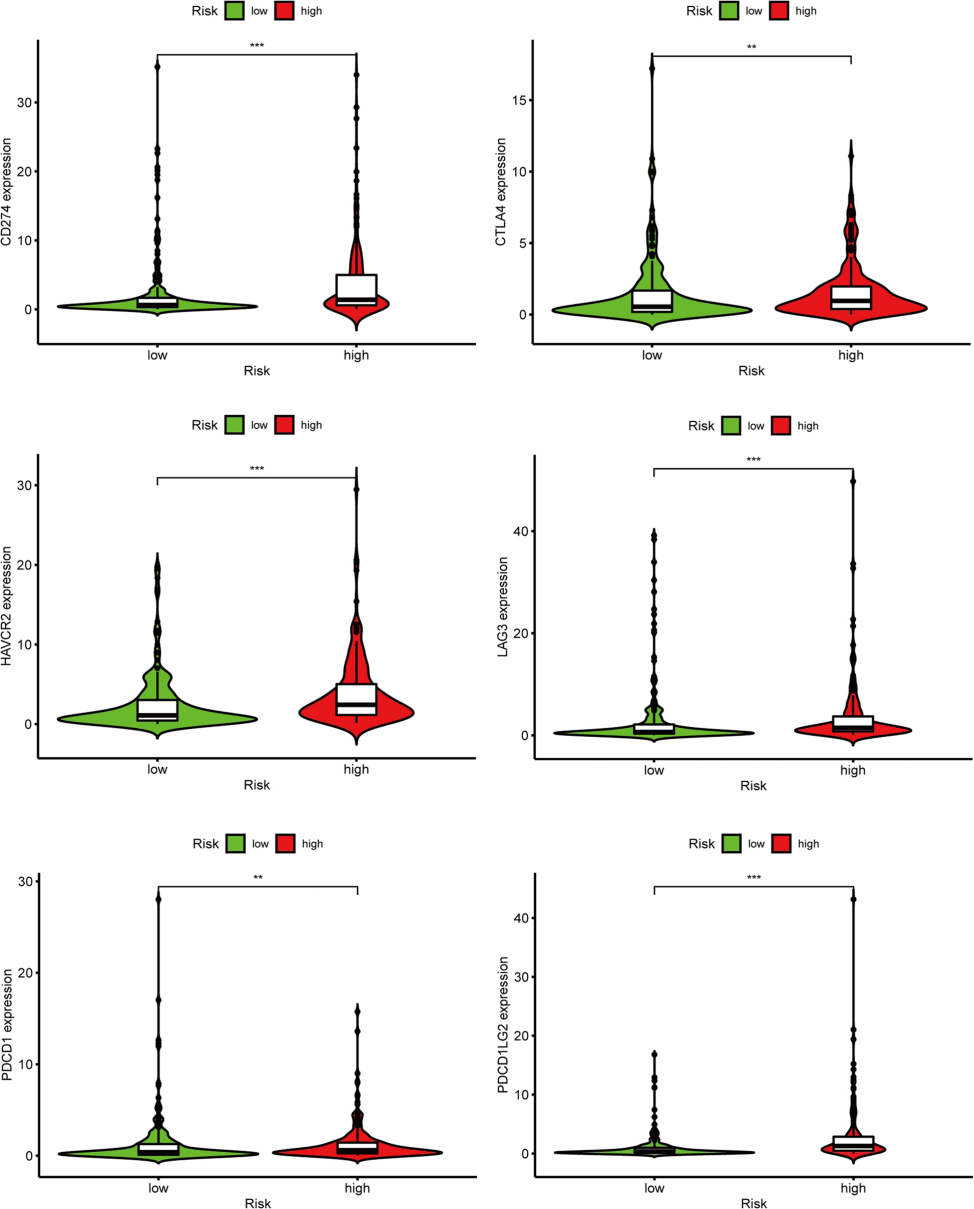
The relationship between prognostic signature and immune checkpoints

The relationship between the signature and immune infiltration was displayed in the heatmap according to the analyses of TIMER, CIBERSORT, CIBERSORT-ABS, XCELL, QUANTISEQ, EPIC, and MCP-counter (Figure 12). The result of CIBERSORT indicated that the proportions of CD8+ T cells, Tregs, and activated dendritic cells were higher in the low-risk group, whereas M0 macrophages and M2 macrophages had higher proportions in the high-risk groups (Supplemental Figure 3). Given the significance of checkpoint inhibitor immunotherapies, we also investigated the correlation between risk score and key immune checkpoints (PDCD1, PD-L1, LAG3, HAVCR2, TIGIT, and CTLA4). We found a prominent difference in the expression of PDCD1, PD-L1, LAG3, HAVCR2, TIGIT, and CTLA4 between the two groups of patients. In addition, PD-1, PD-L1, LAG3, HAVCR2, TIGIT, and CTLA4 were elevated in the high-risk groups, suggesting an immunosuppressive and exhausted phenotype in the high-risk groups (Figure 13).

### Identification of small molecule drugs

We acquired the 8 most potential small molecule drugs on the basis of CRs through the CMAP database. They were phenoxybenzamine, pyrvinium, trichostatin A, ionomycin, methylbenzethonium chloride, rottlerin, lanatoside C, and monorden (Table 2).

**Table 2:** The 8 small molecule drugs of CMP dataset analyses results

CMAP names	enrichment	*p* value	*n*	percent non-null
phenoxybenzamine	-0.973	0.00006	3	100%
pyrvinium	-0.922	0.00004	4	100%
trichostatin A	-0.88	0.00000	55	100%
ionomycin	-0.873	0.00411	3	100%
rottlerin	-0.841	0.00803	3	100%
lanatoside C	-0.838	0.00855	3	100%
monorden	-0.81	0.00056	5	100%
Methylbenzethonium chloride	-0.859	0.00563	3	100%

### TIMER analysis

TIMER database was applied to explore the relationship between immune cells and 11 prognostic CRs. The results showed SETBP1, ZHX3, and TCF4 were positively associated with multiple immune cells such as CD8+ T cells, CD4+ T cells, macrophage, neutrophil, and dendritic cells. HMGA1 was negatively correlated with macrophage and positively correlated with CD8+ T cells, neutrophils, and dendritic cells. SETD7 and DUSP1 were positively associated with CD8+ T cells, macrophage, neutrophils, and dendritic cells. ARID3A and RAC3 were positively related to macrophage. Meanwhile, RAC3 also was negatively related to dendritic cells. SATB1 was negatively connected with CD8+ T cell and dendritic cells while positive related to macrophage and B cell. RCOR2 was negatively connected with CD8+ T cells, neutrophils, and dendritic cells. CBX7 was positively related to CD4+ T cells, macrophage, and B cells while negatively in connection with B cell, neutrophils, and dendritic cells (Supplemental Figure 1 and Figure 2).

### Drug sensitivity analysis

**Figure 14:**
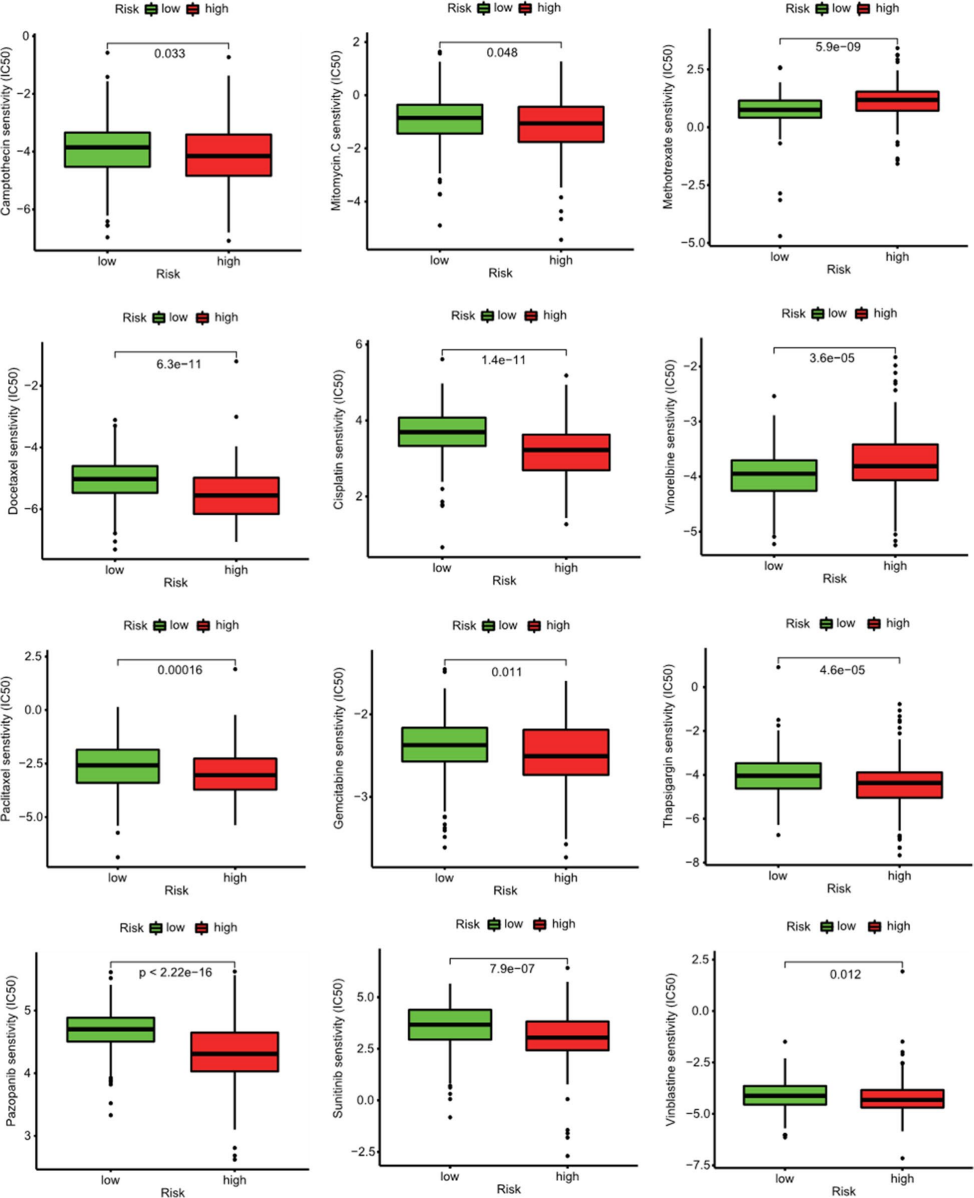
Drug sensitivity analysis

To advance the therapeutic effect of patients with BLCA, we further investigated the sensitivity difference of common chemotherapy drugs among two groups. The results of GDSC database analysis indicated that IC50 values of drugs including Camptothecin, Mitomycin C, Thapsigargin, Gemcitabine, Pazopanib, Docetaxel, Sunitinib, Cisplatin, and Vinblastine were higher in patients of the high-risk group than those of the low-risk group, which suggested that patients in the high-risk group were much more sensitive to these drugs (Figure 14). While IC50 values of Methotrexate and Vinorelbine were much lower in patients of the low-risk group than those of the high-risk group, suggesting that patients in the low-risk group were much more sensitive to Methotrexate and Vinorelbine.

## Discussion

Several diagnostic and prognostic biomarkers, including mRNAs, miRNAs, lncRNAs, and circRNAs, have been identified for cancers [29–32]. Risk models with high prognostic accuracy were identified as potential prognostic biomarkers based on RNA-binding proteins in many cancer types [33–35]. Prognostic indicators based on long non-coding RNAs have been reported in several pieces of researches such as immune-related lncRNAs [36, 37] and autophagy-related lncRNAs [38, 39], which also showed well performance in predicting the overall survival of cancer patients. ROC curves showed that the AUC of circ_0004826 was 0.790, which suggested the availability of distinguishing cancer tissues and normal tissues [40]. A risk score model based on three miRNAs (miR-126-3p, miR-143-5p, and miR-1275) was identified as a potential biomarker for GC prognosis [41]. A growing number of studies have demonstrated that CRs exert various functions in BLCA tumorigenesis. However, few studies have comprehensively analyzed CRs to investigate the clinical significance of CRs in BLCA.

In the present study, we first screened 91 CRs that were differentially expressed between BLCA tissues and normal bladder tissues in the TCGA database. We systematically explored the biological pathways and constructed PPI networks for 91 CRs. Then, we identified 11 CRs related to BLCA prognosis through performing univariable and lasso-penalized Cox regression analyses. Based on these 11 CRs, we established and validated a risk model associated with outcome. Survival and ROC analyses have shown that the good predictive ability of the model. Finally, the results of univariable and multivariable Cox analyses have indicated that the risk score based on 11 CRs was an independent prognostic indicator for BLCA patients. Furthermore, we also found that the signature was closely related to immune cells infiltration and 8 small molecule drugs were identified for the treatment of patients with BLCA.

GO analyses uncovered that CRs were mainly related to process terms, such as covalent chromatin modification, histone modification, DNA replication, and chromatin binding. The result of pathway enrichment analyses indicated that 91 CRs were mainly involved in the cell cycle, apoptosis, p53 signaling pathway, MAPK signaling pathway, and FoxO signaling pathway. These pathways were strongly linked to malignant phenotypes of various malignancies, which implied that CRs might play critical roles in the tumorigenesis and progression of cancer. Additionally, TIMER database analyses showed that 11 prognostic CRs of the model were related to immune cell infiltration, which revealed that CRs might regulate cancer progression by influencing immune infiltration.

Zinc finger and homeobox 3 (ZHX3), a ubiquitous transcriptional repressor, has been reported to participate in various cancers, including bladder cancer [42], breast cancer [43], gastric cancer [44] and renal cancer [45]. Overexpression of ZHX3 was positively related to worse clinical characteristics including N stage and recurrence. A high ZHX3 expression was an independent factor, which indicated an unfavorable prognosis in BLCA patients. ZHX3 exerted an oncogenic role in BLCA by inhibiting the RGS2/RhoA pathway. TCF4, as an important regulator of epithelial-mesenchymal transition and downstream of the Wnt/β-catenin signaling pathway, has been reported to be involved in cancer metastasis [46]. SET binding protein 1 (SETBP1), known as PP2A phosphatase activity inhibitor, was implicated in cancer pathogenesis such as leukemic malignancies [47], colorectal cancer [48], lung cancer [49], and breast cancer [50]. Under-expression of SETBP1 promoted the proliferation and invasion of NSCLC cells through activating the ERK1/2 signaling pathway. SET domain containing 7(SETD7), a histone lysine methyltransferase, has been reported to be dysregulated in various cancers. However, several pieces of research have demonstrated that SETD7 was a tumor suppressor gene in BLCA [51]. High-mobility group A1(HMGA1) deregulated in a variety of cancers including breast cancer [52], lung cancer [53], cervical cancer [54], and bladder cancer [21]. Our previous study indicated that HMGA1 was significantly elevated in BLCA, and HMGA1 silencing could suppress tumorigenic phenotypes of BLCA cells by inhibiting the miR-221/TP53INP1 axis. Dual-specificity protein phosphatase 1 (DUSP1) was involved in proliferation, autophagy, and apoptosis through regulating MAPK and SAPK/JNK signaling pathways. DUSP1 was upregulated in BLCA tissues and inhibited cancer cells proliferation [55]. The function of Chromobox 7(CBX7) in cancer remains controversial. Some studies have shown that CBX 7 exerted anti-cancer function in many cancers. Several studies also found that CBX 7 might play an oncogenic role in multiple cancers. AT-rich interactive domain 3A (ARID3A) was considered as an independent prognostic predictor for several cancers. ARID3A facilitated the malignant phenotypes through upregulating AURKA in colorectal cancer [56]. Rac family of small GTPase 3 (RAC3), one of the Rho GTPase family members, has been reported that overexpression of RAC3 emerged in several cancers. RAC3 was also embroiled in cancer cell proliferation and aggressiveness [57, 58]. Furthermore, overexpression of RAC3 could enhance cell proliferation, migration, and invasion by activating JAK/STAT signaling in BLCA [59]. Special AT-rich sequence-binding protein-1 (SATB1) might have diverse functions in cancer dependent on tumor type and stage [60]. One study found that SATB1 knockdown in BLCA HTB-9 cells promoted cell growth and decreased sensitization to cisplatin. Meanwhile, SATB1 silencing in BLCA HTB-5 cells reduced cell proliferation and enhanced sensitization to cisplatin [61]. Another study reported that SATB1 accelerated the malignant progression of BLCA via inducing epithelial-mesenchymal transition (EMT) [62]. RCOR2 could activate LSD1, recently identified as a potent inhibitor of anti-tumor immunity [63]. RCOR2 was positively correlated with ERV, IFN, and ISG. Upregulation of RCOR2 in tumors provided a selective advantage by LSD1-mediated immune evasion [64].

The results of GSEA analysis indicated that CR-based signature was mainly implicated in cancer- and metabolism-related pathways, such as pathways in cancer, TGF-β signaling pathway, oxidative phosphorylation, fatty acid metabolism, and MAPK signaling pathway. Hence, CR-based signature has the predictive ability of prognosis in patients with BLCA and might function as a crucial role in BLCA biology.

CD8+T cells suggested a favorable prognosis and better response from pembrolizumab, which was consistent with our results that the proportions of CD8+ T cells were higher in the low-risk group. Patients with BLCA in the high-risk groups had the higher expression of PDCD1, PD-L1, Tim-3, CTLA4, TIGIT, and LAG3 than those in the low-risk groups, which indicated that the unfavorable prognosis of BLCA patients in high-risk groups might be owing to the immunosuppressive microenvironment. Moreover, BLCA patients in the high-risk groups might benefit from checkpoint inhibitor immunotherapies. In addition, we also found that BLCA patients in the high-risk group might benefit from the treatments of Camptothecin, Pazopanib, Docetaxel, Mitomycin C, Thapsigargin, Gemcitabine, Sunitinib, Cisplatin, and Vinblastine, and BLCA patients in the low-risk group might benefit from the treatments of Methotrexate and Vinorelbine.

This study also had some shortcomings. The mechanisms on how CRs regulated the biological behavior of BLCA cells should be verified by experiments. In addition, a multicenter clinical cohort should be used to verify the practicability of the prognostic model.

## Conclusions

In conclusion, we identified differentially expressed CRs that might involve in carcinogenesis and progression of BLCA. CRs have important values in predicting the outcome of BLCA patients and targeting CRs showed the potential application as an effective treatment of bladder cancer. Our study also should be validated by further research.

## Supplementary Information

**Additional file 1: Supplemental Figure 1** Immune cells infiltration of 11 CRs by TIMER database.

**Additional file 2: Supplemental Figure 2** Immune cells infiltration of 11 CRs by TIMER database.

**Additional file 3: Supplemental Figure 3** The difference of immune cells between two groups by CIBERSORT.

## Data Availability

The raw data of this study are derived from the TCGA database (https://portal.gdc.cancer.gov/) and GEO data portal (https://www.ncbi.nlm.nih.gov/geo/), which are publicly available databases.

## Abbreviations

BLCA: Bladder Cancer
CR: Chromatin regulator
TCGA: The Cancer Genome Atlas
GEO: Gene Expression Omnibus
KEGG: Kyoto Encyclopedia of Genes and Genomes
FDR: False discovery rate
FC: Fold change
GO: Gene ontology
