# Supplementary figures and images for: Identification of a chromatin regulator signature and potential candidate drugs for bladder cancer
Source: Hereditas. 2022 Feb 7;159:13. doi: 10.1186/s41065-021-00212-x (PMC8819906; doi:10.1186/s41065-021-00212-x)

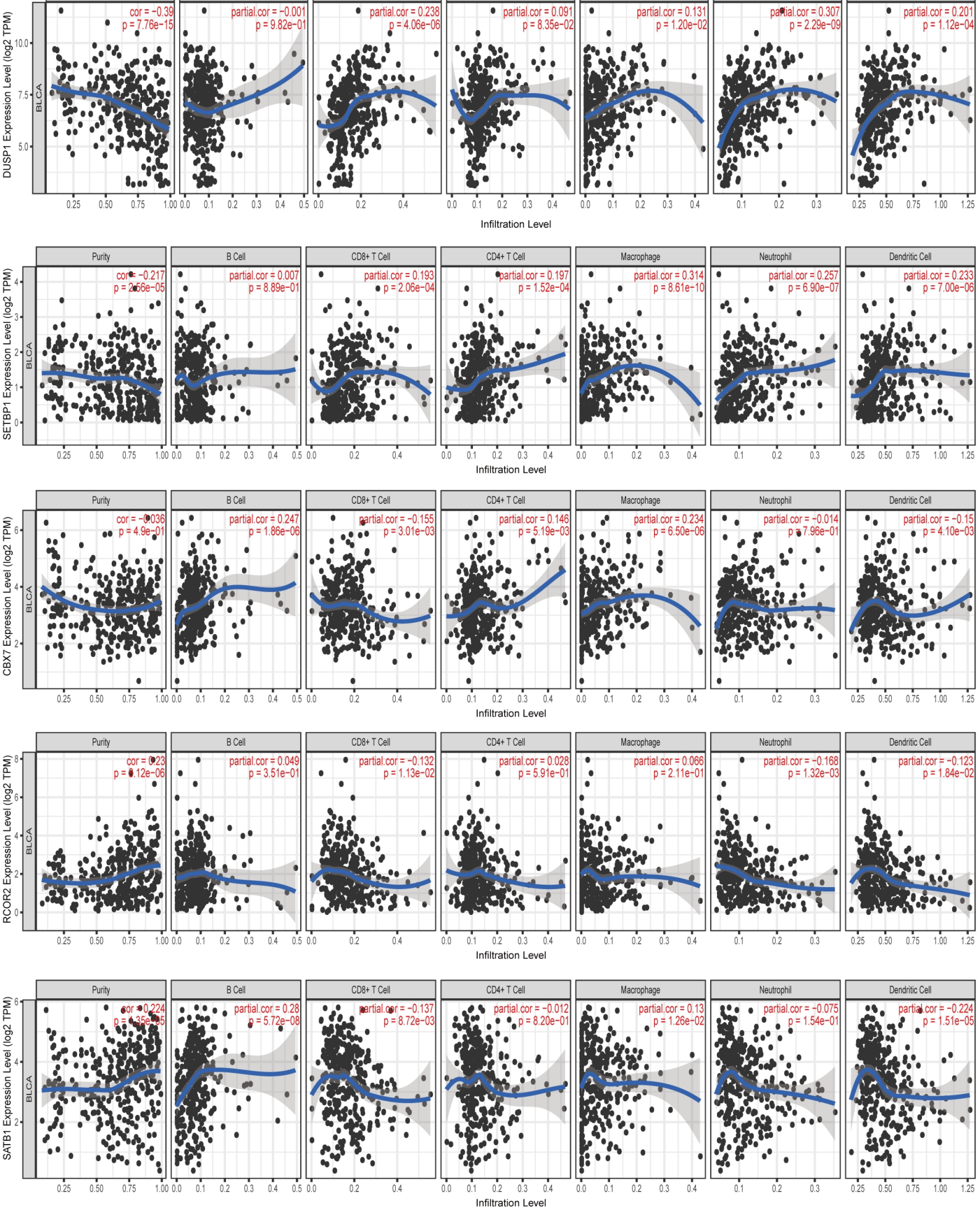

Supplement: Supplementary file 1 — Additional file 1: Supplemental Figure 1 Immune cells infiltration of 11 CRs by TIMER database. [file 41065_2021_212_MOESM1_ESM.pdf]

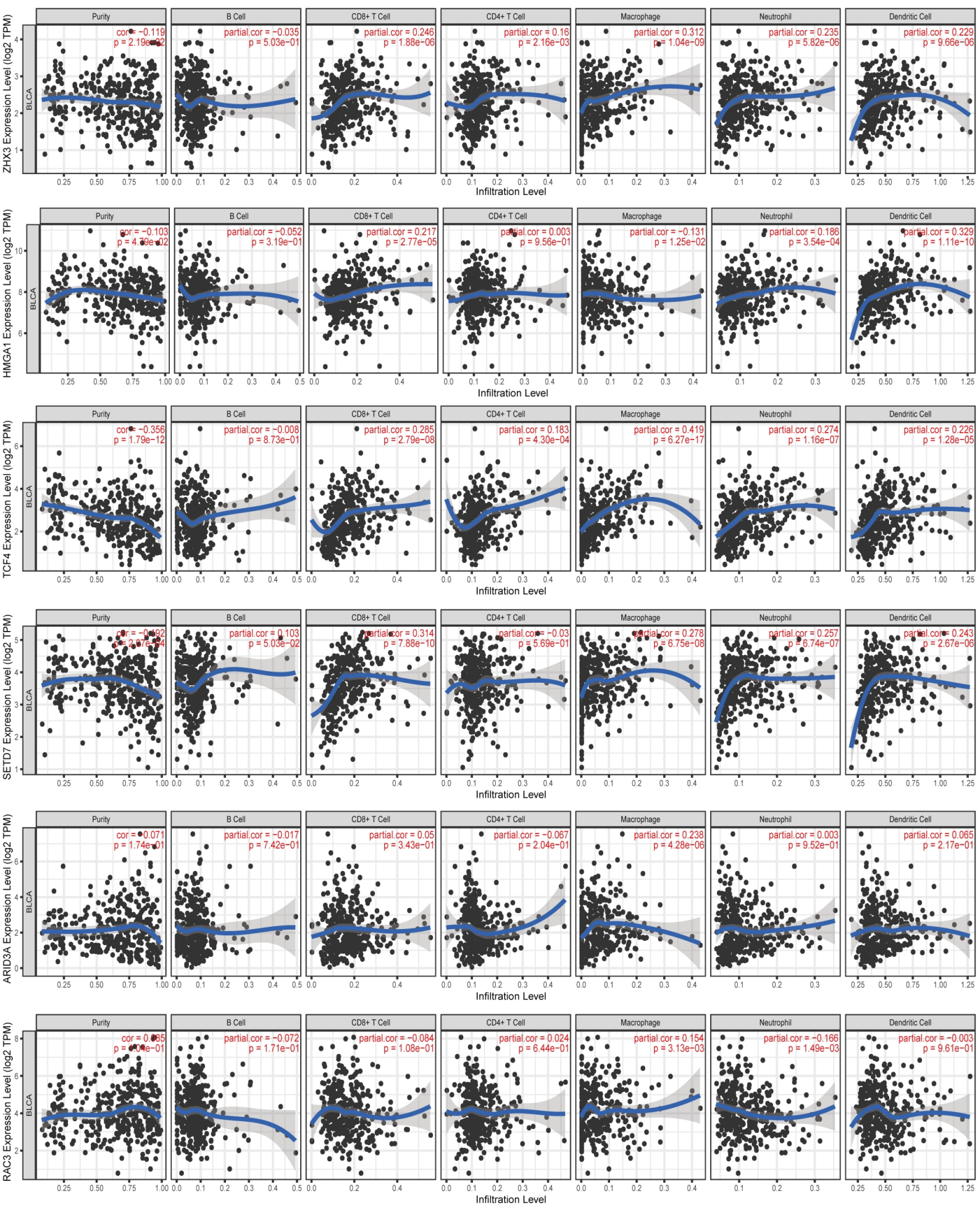

Supplement: Supplementary file 2 — Additional file 2: Supplemental Figure 2 Immune cells infiltration of 11 CRs by TIMER database. [file 41065_2021_212_MOESM2_ESM.pdf]

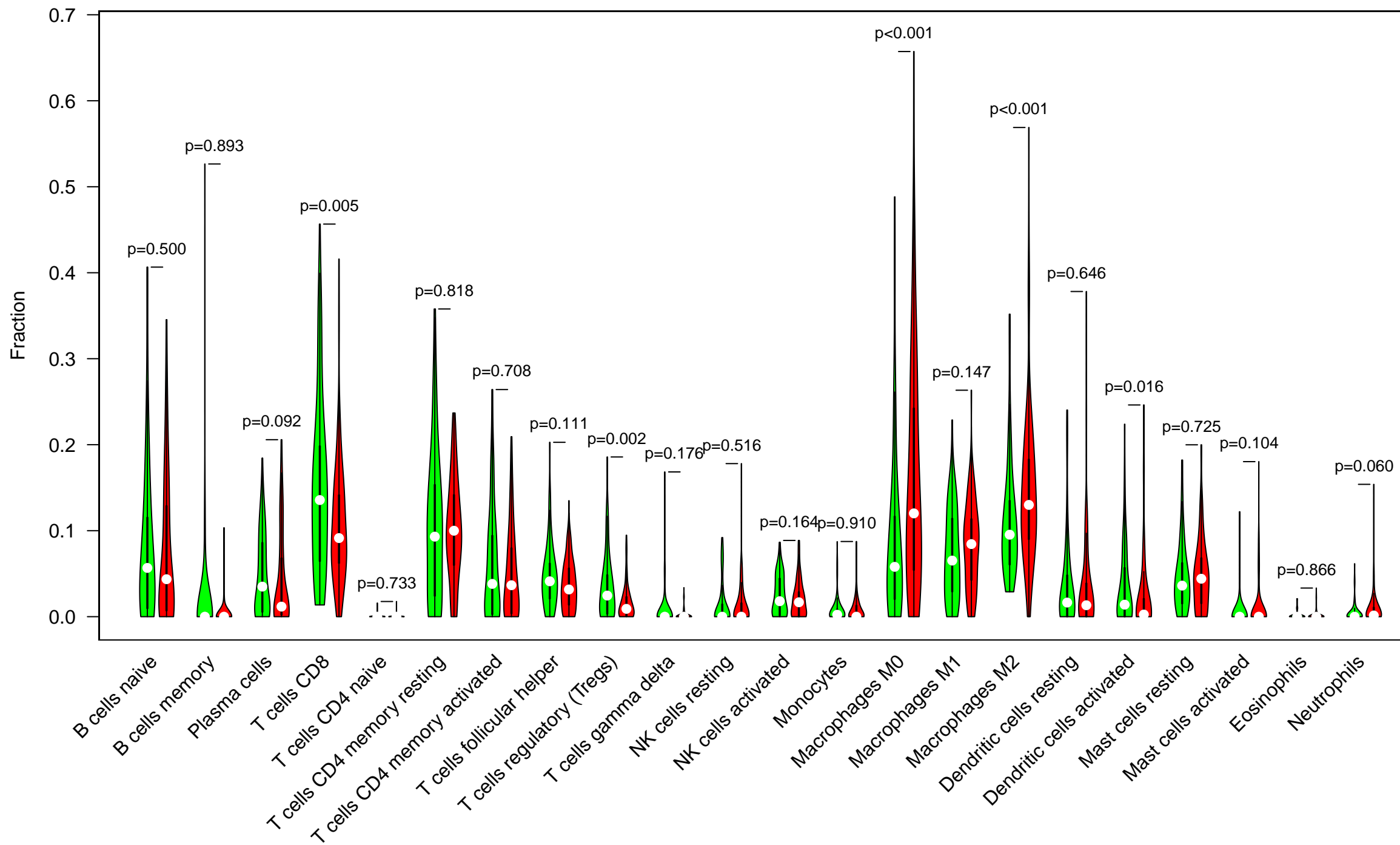

Supplement: Supplementary file 3 — Additional file 3: Supplemental Figure 3 The difference of immune cells between two groups by CIBERSORT. [file 41065_2021_212_MOESM3_ESM.pdf]
